# Inhibition of Rab27a and Rab27b Has Opposite Effects on the Regulation of Hair Cycle and Hair Growth

**DOI:** 10.3390/ijms21165672

**Published:** 2020-08-07

**Authors:** Kyung-Eun Ku, Nahyun Choi, Jong-Hyuk Sung

**Affiliations:** 1 College of Pharmacy, Yonsei Institute of Pharmaceutical Sciences, Yonsei University, Incheon 21983, Korea; ku88120@yonsei.ac.kr; 2 STEMORE Co. Ltd., Incheon 21983, Korea; nh147837@gmail.com

**Keywords:** Rab27a, Rab27b, anagen induction, hair cycle, hair growth, outer root sheath cells, dermal papilla cell, growth factors

## Abstract

Rab27a/b are known to play an important role in the transport of melanosomes, with their knockout causing silvery gray hair. However, the relationship between Rab27a/b and hair growth is not well known. To evaluate the role of Rab27a/b in hair cycle, we investigated the expression of Rab27a/b during hair cycling and human outer root sheath (hORS) cells. The expression of Rab27a in ORS cells was mainly detected at the anagen, whereas expression of Rab27b in ORS, and epidermal cells was strongly expressed at the telogen. Additionally, Rab27a/b were expressed in the Golgi of hORS cells. To evaluate the role of Rab27a/b in hair growth, telogen-to-anagen transition animal and vibrissae hair follicles (HFs) organ culture models were assayed using Rab27a/b siRNAs. The knockdown of Rab27a or Rab27b suppressed or promoted hair growth, respectively. These results were also confirmed in human dermal papilla cells (hDPCs) and hORS cells, showing the opposite mitogenic effects. Moreover, Rab27b knockdown increased the expression levels of various growth factors in the hDPCs and hORS cells. Overall, the opposite temporal expression patterns during hair cycling and roles for hair growth of Rab27a/b suggested that Rab27a/b might regulate the hair cycle. Therefore, our study may provide a novel solution for the development of hair loss treatment by regulating Rab27a/b levels.

## 1. Introduction

Alopecia is a hair loss disease that occurs for a variety of reasons, including stress, aging, and hormonal abnormalities [1]. Hair loss treatment varies depending on the cause and unique characteristics of the hair loss. For example, in the case of male pattern hair loss, oral medications, such as finasteride, or drugs applied to the scalp, such as minoxidil and ketoconazole, have been used primarily. In the case of alopecia areata, steroids have often been used to treat hair loss [2]. There are also treatments for hair loss in which healthy hair is transplanted into the hair loss area [3]. However, trends in today’s hair loss treatments and clinical studies appear to be more focused on biological approaches [4]. These trends show approaches that promote hair growth by promoting the proliferation of hair follicles (HFs) constituent cells or by extending or promoting the anagen phase of the hair cycle [5,6,7].

Rab27, a member of the Rab subfamily of small GTPases, is largely classified into Ras-related protein Rab-27A (Rab27a) and Ras-related protein Rab-27B (Rab27b) [8,9]. These two proteins have a structural similarity of approximately 70% and are primarily involved in vesicle-mediated transport in various cells [10,11,12]. However, despite their structural and functional similarities, different physiological results have been demonstrated depending on the various effectors binding these two proteins. According to recent results, both Rab27a and Rab27b regulate the release of secretory lysosomes from various cell types, including mast cells. However, these studies revealed that Rab27A and Rab27B perform different functions in specific types of secretions even within the same cell type by the interaction of different Rab27 effectors, such as Slp, Slac2, and Munc13-4, [13,14]. In addition, they have been reported to function differently, even in the same cell [14,15,16]. An example that demonstrates the different functions of these two proteins is Griscelli syndrome type II [17]. Griscelli syndrome type II is a disease caused by knockout of Rab27a, resulting in silvery gray hair and immunodeficiency owing to the disruption of transport of melanosomes and regulation of secretion in other immune cells, including neutrophils and cytolytic T lymphocytes [18,19,20]. However, Rab27b has been upregulated in human Griscelli syndrome type II melanocytes [21].

To date, the association of Rab27 proteins with hair growth has not been well known, except for the silvery gray hair symptoms of Griscelli syndrome type II disease [18,22]. However, there is much evidence showing a strong relationship between hair color and hair growth. Usually, when melanin is produced well in hair, hair growth also occurs at the same time [23,24]. For example, hair color becomes darker in adolescence and young adulthood than in childhood due to androgens and estrogens, which also regulate hair growth [23,25]. In addition, there is also evidence that melanin is produced better in the anagen phase, the active growth phase of HFs, than in the catagen and telogen phases [24].

Therefore, this study investigated whether Rab27a and Rab27b have a hair growth promoting effect and whether they play a key role in hair cycle regulation. The expression of Rab27a and Rab27b during hair cycling and in human outer root sheath (hORS) cells was examined. The hair growth effect of Rab27a/b was investigated by telogen-to-anagen transition model and vibrissae HFs organ culture using siRNA for Rab27a/b. The mitogenic effect and the expression levels of growth factors were examined in human dermal papilla cells (hDPCs) and hORS cells using siRNA for Rab27a/b.

## 2. Results

### 2.1. Rab27a and Rab27b Are Primarily Expressed in the ORS Cells

**Figure 1:**
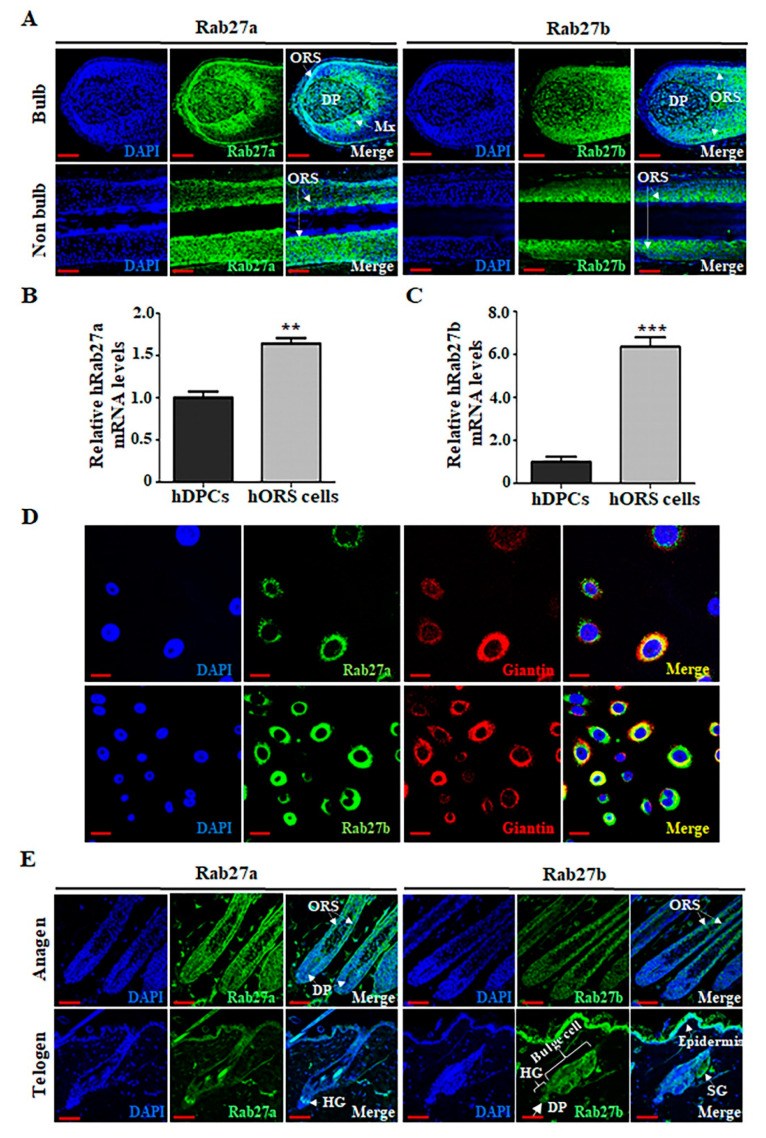
Rab27a and Rab27b are mainly distributed in the outer root sheath (ORS) cells of the hair follicles (HFs) during the anagen phase, both of which are expressed in the Golgi apparatus of hORS cells. (**A**) In human HFs at the anagen phase, Rab27a is mainly distributed in hORS cells and matrix cells (Mx) and is also slightly distributed in hDPCs in the bulb region. In addition, Rab27a was mainly distributed in hORS cells in non-bulb regions. And Rab27b was also mainly distributed in ORS cells in both bulb regions and non-bulb regions. (**B**) The expression level of hRab27a mRNA was increased 1.6-fold in hORS cells compared with that in hDPCs. (**C**) The expression level of hRab27b mRNA was increased 6.4-fold in hORS cells compared with that in hDPCs. (**D**) Both Rab27a and Rab27b were expressed in the Golgi apparatus in hORS cells. (**E**) In mouse HFs at anagen phase, mouse Rab27a/b are mainly distributed in the ORS cells of HFs. However, during the telogen period, mouse Rab27a was mainly expressed in hair germ (HG), and mouse Rab27b is strongly expressed in the epidermis and epidermal compartment, including bulge, HG, and sebaceous gland (SG). hDPCs; human dermal papilla cells, hORS cells; human outer root sheath cells, Mx; matrix cells. Scale bars indicate 50 µm (**A**,**E**) and 20 µm (**D**). ** *p* < 0.01, *** *p* < 0.001 using Student’s *t* test. Three independent experiments were conducted. All error bars indicate S.E.M.

We first examined where Rab27a or Rab27b is expressed during human and mouse hair cycling. They are mainly distributed in the ORS cells of HFs in the anagen phase. In human HFs at the anagen phase, Rab27a is mainly distributed in hORS cells and matrix cells and was also slightly distributed in hDPCs in the bulb region. In addition, Rab27a was mainly distributed in hORS cells in non-bulb regions. In addition, Rab27b was mainly distributed in hORS cells in both bulb regions and non-bulb regions (Figure 1A). We also compared the expression of Rab27a or Rab27b in hDPCs and hORS cells. The expression level of Rab27a in hORS cells was higher than in hDPCs (Figure 1B). Rab27b also showed higher expression in hORS cells compared with hDPCs (Figure 1C). When the subcellular localization of Rab27a and Rab27b was examined in hORS cells, both Rab27a and Rab27b were strongly expressed in hORS cells and especially colocalized with the Golgi marker (giantin) (Figure 1D), suggesting that both Rab27a and Rab27b were expressed in the Golgi of hORS cells. Similarly, Rab27a/b are mainly distributed in the ORS cells of mouse HFs at the anagen phase. Rab27a was mainly expressed in hair germ (HG), which will be ORS cells as the HF grows, and Rab27b is also strongly expressed in the epidermis and epidermal compartment, including bulge, HG, and sebaceous gland (SG), at the telogen phase (Figure 1E).

### 2.2. Inhibition of Rab27a or Rab27b Revealed Opposite Effects for Hair Growth

**Figure 2:**
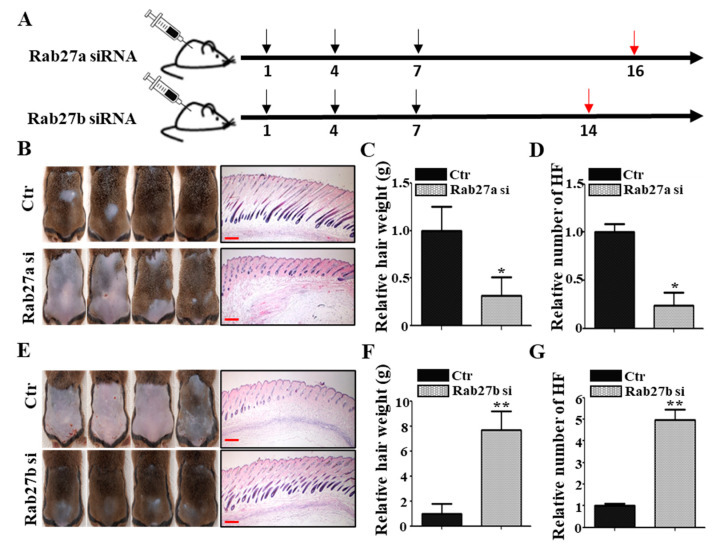
Rab27a and Rab27b have opposite effects in mice. (**A**) In vivo, Rab27a siRNA was injected into the mouse dorsal skin once every 3 days for a total of three times, and the hair and dorsal tissue of the mice were harvested after 16 days. Rab27b siRNA was injected into the mouse dorsal skin once every 3 days for a total of three times, and the hair and dorsal tissue were harvested after 14 days. (**B**–**D**) Knockdown of Rab27a inhibited telogen-to-anagen transition evidenced by hair weight and the number of hair follicles compared with the control. (**E**–**G**) In contrast, knockdown of Rab27b promoted telogen-to-anagen transition evidenced by hair weight and the number of hair follicles compared with the control. Ctr; control, si; siRNA. Black arrows indicate the day of siRNA injection. Red arrows indicate that samples were harvested that day. Scale bars indicate 200 µm (**B**). * *p* < 0.05, ** *p* < 0.01, using Student’s *t* test. *n* = 4 per group. Three independent experiments were conducted. All error bars indicate S.E.M.

To investigate whether Rab27a or Rab27b has a role for hair growth, the knockdown effect of Rab27a and Rab27b was examined by telogen-to-anagen transition model using 6-week-old male C3H mice. The knockdown of Rab27a or Rab27b was confirmed in hORS cells by quantitative real-time reverse transcription polymerase chain reaction (qRT-PCR) (Figure S1A,B). Negative control for siRNA (Ctr), Rab27a siRNA, or Rab27b siRNA was injected into the shaved C3H mice once every 3 days (three times in total). Skin samples for Rab27a siRNA and Rab27b siRNA results were harvested at 16 and 14 days after injection, respectively (Figure 2A). The injection of Rab27a siRNA significantly inhibited the telogen-to-anagen transition evidenced by decreased dorsal hair weight and HF number compared with the negative control (Figure 2B–D). In addition, inhibition by Nexinhib20 (Rab27a inhibitor) also showed the same results (Figure S2E) and produced brighter and thinner hair than the control (Figure S2F,G). Conversely, the injection of Rab27b siRNA significantly promoted the telogen-to-anagen transition in C3H mice. The hair weight and HF number in the Rab27b siRNA-injected group were increased compared with the control (Figure 2E–G). Collectively, knockdown of Rab27a suppressed telogen-to-anagen transition, whereas knockdown of Rab27b promoted telogen-to-anagen transition, suggesting that these proteins have an opposite role for hair growth. 

### 2.3. Inhibition of Rab27a or Rab27b Revealed the Opposite Effect for Mouse Vibrissae Growth

**Figure 3:**
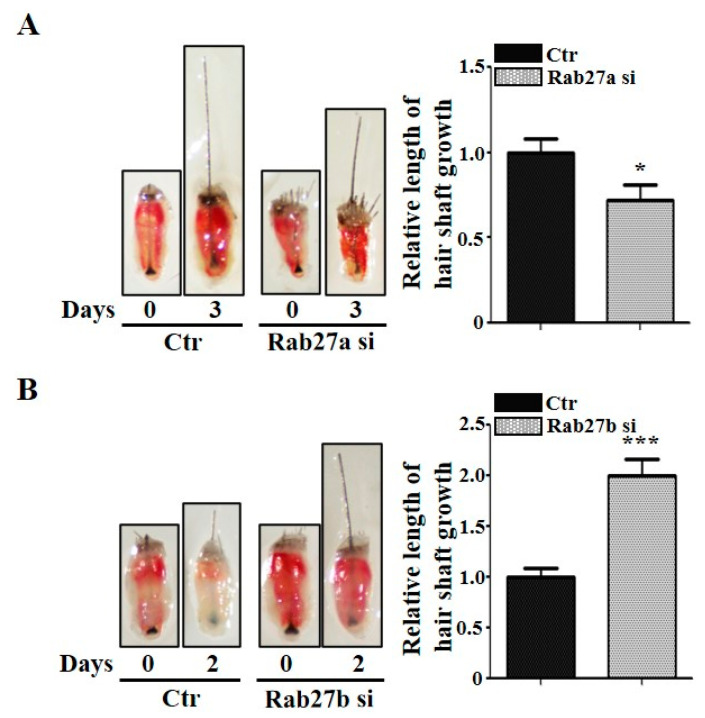
Rab27a and Rab27b have opposite effects in mouse vibrissae. (**A**) When Rab27a siRNA was treated with mouse vibrissae for 3 days, it suppressed the growth of the hair shaft compared with the control. (**B**) On the other hand, inhibition by Rab27b siRNA promoted the hair shaft growth of mouse vibrissae and grew nearly twice as much as the control in 2 days. Ctr; control, si; siRNA, DP; dermal papilla. * *p* < 0.05, *** *p* < 0.001 using Student’s *t* test. *n* = 10 per group. Three independent experiments were conducted. All error bars indicate S.E.M.

We further investigated whether knockdown of Rab27a or Rab27b regulates the growth in the mouse vibrissae organ culture model. The knockdown of Rab27a inhibited the growth of the vibrissae hair shaft compared with the control (Figure 3A; Figure S3A). The suppression of Rab27a by Nexinhib20 also showed a decreased growth rate in the mouse vibrissae hair shaft compared with the control (Figure S2D). On the other hand, the knockdown of Rab27b significantly increased the length of the mouse vibrissae hair shaft compared with the control (Figure 3B; Figure S3B). These data correspond to the results shown in Figure 1.

### 2.4. Inhibition of Rab27a or Rab27b Showed Opposite Mitogenic Effects in hORS Cells and hDPCs 

**Figure 4:**
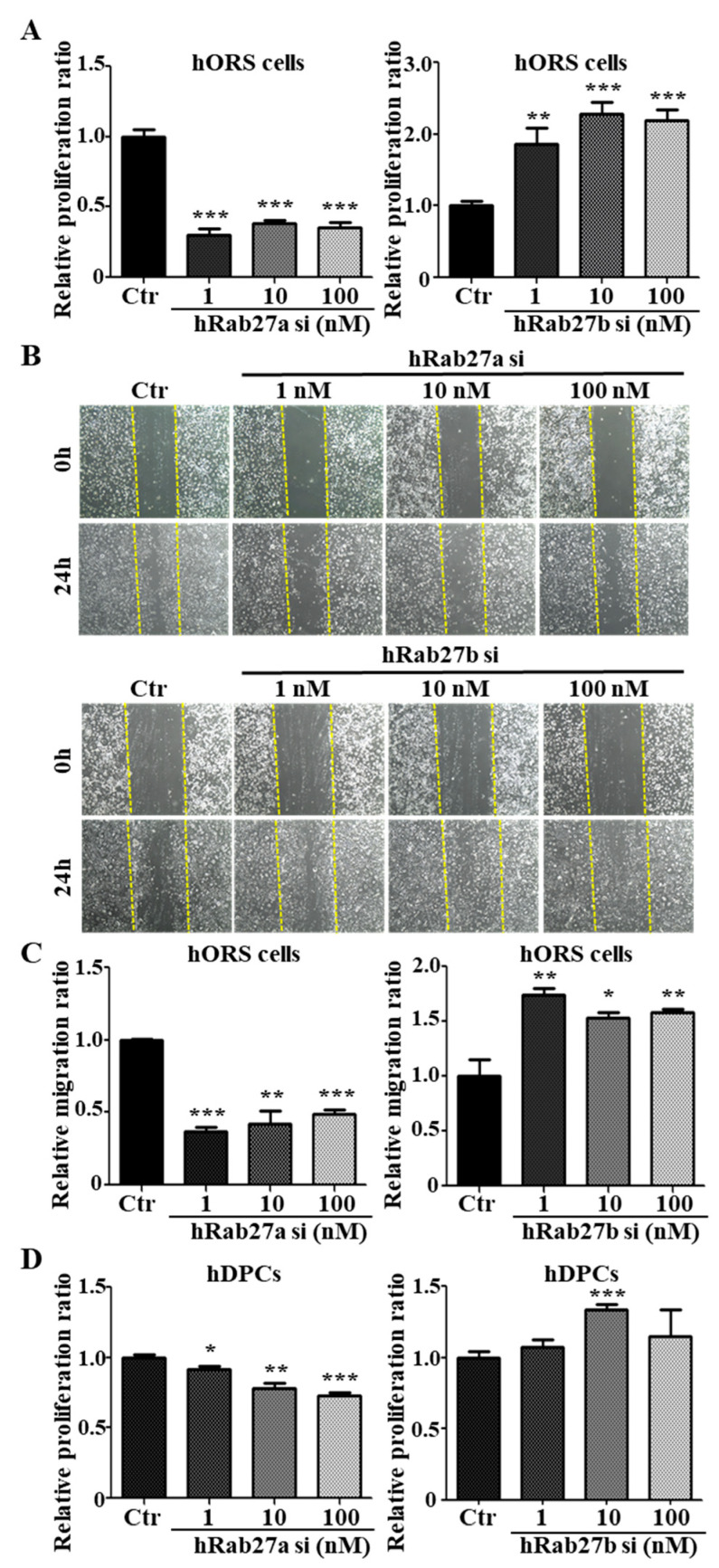
Rab27a and Rab27b have opposite effects on the proliferation and migration of hORS cells and hDPCs. (**A**) The knockdown of hRab27a decreased the proliferation of hORS cells by up to 70% compared with the control. On the other hand, knockdown of hRab27b increased the proliferation of hORS cells by up to 230% compared with the control. (**B**) The knockdown of Rab27a in hORS cells for 24 h decreased the migration of hORS cells, whereas knockdown of Rab27b for 24 h increased the migration of hORS cells. (**C**) The migration assay results were quantified. (**D**) Knockdown of hRab27a decreased the proliferation of hDPCs in a concentration-dependent manner. On the other hand, knockdown of hRab27b increased the proliferation of hDPCs and showed the highest effect at a concentration of 10 nM. hDPCs; human dermal papilla cells, hORS cells; human outer root sheath cells, Ctr; control, si; siRNA. * *p* < 0.05, ** *p* < 0.01, *** *p* < 0.001 using Student’s *t* test. Three independent experiments were conducted for all data points. All error bars indicate S.E.M.

To investigate whether Rab27a or Rab27b regulated the mitogenic effect in hORS cells, we performed a proliferation assay and a scratch migration assay using siRNAs for Rab27a and Rab27b. When siRNAs for Rab27a were treated on hORS cells, the proliferation and migration were decreased compared with the control (Figure 4A,B). Nexinhib20 also suppressed the proliferation and migration in hORS cells (Figure S2A,B). On the other hand, when siRNA for Rab27b was treated with 1–100 nM, the proliferation and migration of hORS cells were increased dose dependently (Figure 4A–C).

To examine the effect of Rab27a or Rab27b on the proliferation of hDPCs, we treated Rab27a siRNA or Rab27b siRNA in hDPCs, respectively. Knockdown of Rab27a inhibited the proliferation of hDPCs by up to 27% at 100 nM, and knockdown of Rab27b increased by up to 34% at 10 nM (Figure 4D). When Nexinhib20 was treated on hDPCs, the proliferation was also reduced (Supplemental Figure S2C). All data indicated that knockdown of Rab27a suppressed and knockdown of Rab27b promoted the mitogenic effect in hORS cells and hDPCs.

### 2.5. Rab27b Inhibition Increased the Secretion of Multiple Growth Factors

**Figure 5:**
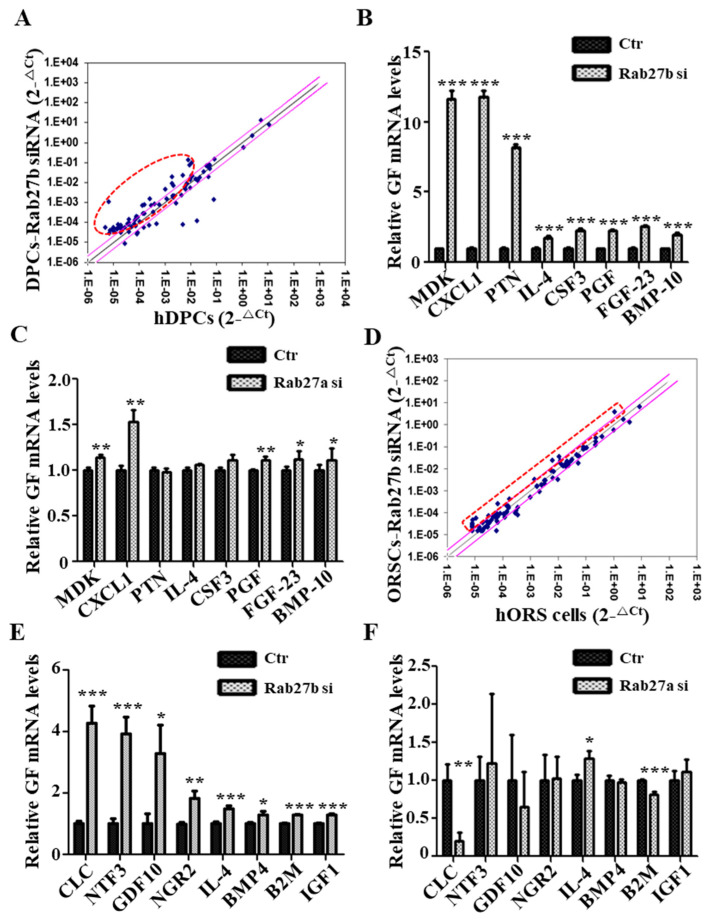
Rab27b inhibition increased expression of various growth factors. (**A**) Scatterplot of qPCR analysis of control and Rab27b siRNA-treated-hDPCs sample. (**B**) Inhibition of Rab27b promoted large production of multiple growth factors, approximately 2- to 12-fold in hDPCs. (**C**) However, Rab27a inhibition had a minor effect on the increase or decrease in expression of these growth factors except *CXCL1* in hDPCs. (**D**) Scatterplot of qPCR array analysis of control and Rab27b siRNA-treated-hORS cells. (**E**) Inhibition of Rab27b promoted the production of multiple growth factors, approximately 1.2- to 4-fold in hORS cells. (**F**) On the other hand, Rab27a inhibition had little effect on the production of most growth factors, except for Charcot-Leyden crystal protein (*CLC*) and β-2-microglobulin (*B2M*). GF; growth factor, si; siRNA. * *p* < 0.05, ** *p* < 0.01, *** *p* < 0.001 using Student’s *t* test. Three independent experiments were conducted for all data points. All error bars indicate S.E.M.

We investigated whether inhibition of Rab27a or Rab27b induces the production of growth factors in hDPCs that play a major role in growth factor production in HFs using qRT-PCR array for approximately 90 human growth factors. The expression levels of several growth factors in Rab27b siRNA-treated hDPCs were markedly upregulated compared with those in the control group (Figure 5A), and the expression of eight genes (i.e., *MDK, CXCL1, PTN, IL-4, CSF3, PGF, FGF23* and *BMP10*) among the upregulated growth factors was confirmed (Figure 5B). However, Rab27a inhibition had a minor effect on the increase or decrease in expression of these growth factors except *CXCL1* in hDPCs (Figure 5C). Likewise, the expression levels of several growth factors (i.e., *CLC, NTF3, GDF10, IL-4, NGR2, BMP4, B2M,* and *IGF1*) in Rab27b siRNA-treated hORS cells were upregulated compared with those in the control group (Figure 5D), and the expression level of the increased growth factors was confirmed by qRT-PCR (Figure 5E). Rab27a inhibition also did not affect the expression of these growth factors, except for *CLC* and *B2M*, in hORS cells (Figure 5F). These results indicated that Rab27b inhibition upregulated the expression of multiple growth factors in hDPCs and hORS cells, thereby promoting hair growth. However, Rab27a has nothing to do with the regulation of these growth factors.

### 2.6. Hair Growth Effect by Upregulated Growth Factors

**Figure 6:**
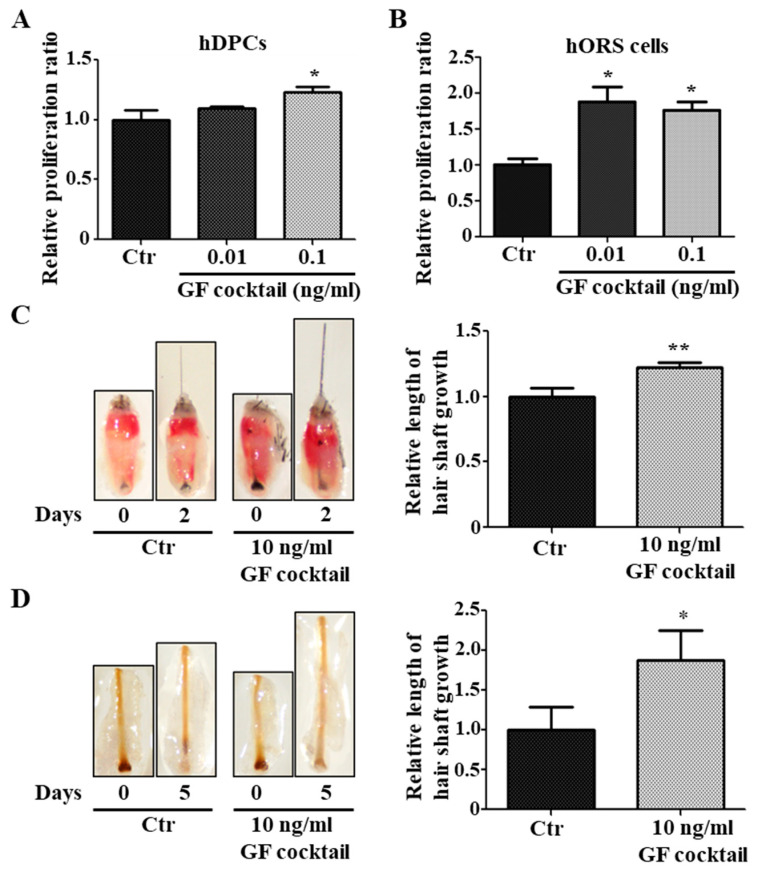
GF cocktail promoted hair growth. (**A**,**B**) GF cocktail increased the proliferation of hDPCs (**A**) and hORS cells (**B**). (**C**) GF cocktail promoted the growth of mice vibrissae hair shaft at a concentration of 10 ng/mL. (**D**) GF cocktail promoted the growth of the length of a pig skin hair shaft at a concentration of 10 ng/mL. GF cocktail; complex of upregulated growth factors in Rab27b siRNA-treated in hDPCs. * *p* < 0.05, ** *p* < 0.01 using ANOVA followed with Tukey’s post hoc test or Student’s *t* test. Three independent experiments were conducted. All error bars indicate S.E.M.

We investigated whether the upregulated growth factors by Rab27b inhibition are responsible for hair growth. Because hDPCs are mainly cells supplying growth factors to HFs, we used a GF cocktail (i.e., MDK, CXCL1, PTN, IL-4, CSF3, PGF, FGF23, and BMP10) that increased by Rab27b knockdown in hDPCs for a test. This growth factor cocktail was treated with hDPCs and hORS cells. As a result, the proliferation of these two types of cells was increased compared with the control (Figure 6A,B). Moreover, the GF cocktail promoted the length of mice vibrissae hair shaft and pig skin hair shaft compared with the control (Figure 6C,D). These results indicated that the upregulated growth factors by Rab27b inhibition in hDPCs and hORS cells can induce hair growth. 

## 3. Discussion

**Figure 7:**
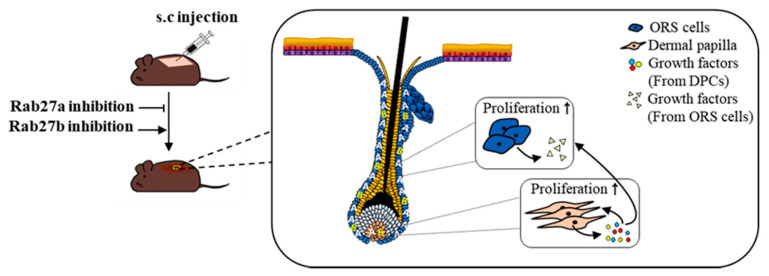
Scheme for regulating hair growth of Rab27a and Rab27b. Rab27a and Rab27b were mainly distributed in ORS cells of HFs. Rab27b inhibition stimulated ORS cells and DPCs and increased the expression of multiple growth factors.

In this study, we presented three major results. First, Rab27a and Rab27b are strongly expressed in the anagen and telogen phase, respectively, and both are mainly expressed in the ORS cells of HFs. Second, knockdown of Rab27a and Rab27b exhibited opposite results on hair growth and mitogenic effects of hDPCs and hORS cells. Third, knockdown of Rab27b increased the expression level of various growth factors in hDPCs and hORS cells. All data suggested that Rab27a and Rab27b play opposite roles for hair growth (Figure 7). In conclusion, this study demonstrated scientific evidence for the applicability of Rab27a/b in the treatment of hair loss. We believe further studies could contribute to the development of alopecia treatments.

To confirm whether Rab27a and Rab27b are involved in hair cycle regulation, we examined their expression during the hair cycle of mice. As a result, Rab27a was mainly expressed in ORS cells in the anagen phase, whereas Rab27b was strongly expressed in the epidermis and epidermal compartment which will be ORS cells as the HF grows, in the telogen phase. These opposite temporal expression levels of Rab27a/b were also confirmed at the mRNA and protein levels (Figure S4). The expression results of Rab27a/b corresponded to the opposite function for hair growth evidenced by telogen-to-anagen transition, HF organ culture, and the mitogenic effects of hDPCs and hORS cells. Given these results, we can deduce that Rab27a and Rab27b may act as an inducer and a suppressor for hair growth, respectively. Therefore, our results suggest that knockdown of Rab27b may be necessary for hair growth.

According to previous studies, Rab27a and Rab27b have structural similarities and are functionally similar in that they are involved in the transport and exocytosis of secretory vesicles in various cell types, including melanocytes, neutrophil, and cancer cells [17,26,27]. However, recent studies have suggested that these two GTPases play different roles both functionally and mechanically, even in the same cell. According to Singh et al., secretory granules in the mast cell are docked at the plasma membrane by Rab27b/Munc13-4 interaction, whereas the Rab27a/Mlph/MyoV interaction regulates F-actin distribution and stability to restrict access of granules to the plasma membrane [14]. In other words, both Rab27a and Rab27b are involved in the transport of vesicles, but each plays a different role in different steps by combining with different effectors. In another example, Ostrowski et al. revealed that Rab27a and Rab27b play different roles in the exosome secretion pathway. According to this study, there were differences in the size and speed of movement of multivesicular endosomes by silencing Rab27a or Rab27b. These results were caused by the binding affinity of Slp4 effector to Rab27a or binding affinity of Slac2b effector to Rab27b [16]. In addition, there have been some cases in which the expressions of Rab27a and Rab27b were opposite, as in Griscelli syndrome type II disease [18,21]. In these previous studies, we can deduce that Rab27a and Rab27b might have functional differences in the cells that make up HFs, resulting in different results in hair cycle regulation and hair growth.

Thornton et al. revealed that dermal papilla cells secrete autocrine growth factors, which are associated with hair growth [28]. Moreover, Fujie et al. and Limat et al. revealed that DPC-derived growth factors promote proliferation and migration of ORS cells [29,30]. Therefore, we constituted the GF cocktail (MDK, CXCL1, PTN, IL-4, CSF3, PGF, FGF23, and BMP10) using the upregulated growth factors of DPCs by Rab27b siRNA treatment. CXCL1 and IL-4 reportedly induced the hair growth, but there is no evidence for other growth factors. Although we did not examine the hair growth promoting effects of each growth factor further, the GF cocktail collectively exhibited the hair growth promoting effects. Therefore, we will further examine the hair growth promoting effect of each growth factor.

## 4. Materials and Methods

### 4.1. Cell Cultures and Chemical Treatment

hDPCs were cultured in Follicle Dermal Papilla Cell Growth Medium with Growth Medium Supplement Mix (PromoCell, Heidelberg, Germany) and 1% Gibco Antibiotic-Antimycotic (Thermo Fisher Scientific, Waltham, MA, USA). hORS cells were cultured in EpiLife medium with EpiLife Defined Growth Supplement (Gibco, New York, NY, USA) and 1% penicillin/streptomycin (Gibco). hDPCs and hORS cells were maintained at 37 °C in a humidified 5% CO_2_ incubator. We used Nexinhib20 (Rab27a inhibitor; Tocris Bioscience, Bristol, United Kingdom). For chemical treatment, hDPCs and hORS cells were seeded, and the Nexinhib20 was treated after 24 h with normal mediums, including 1% penicillin/streptomycin or Gibco Antibiotic-Antimycotic. For cell proliferation assay and migration assay, Nexinhib20 was treated to the cells with different doses (2 or 20 μM) for 3 days, and the cells were then harvested.

### 4.2. siRNA Transfection

The mice used in the experiment were 6-week-old male C3H mice. hDPCs or hORS cells were seeded in a six-well plate at a density of 1.5 × 10^4^ per well, and scramble siRNA, Rab27a siRNA, or Rab27b siRNA was transfected using Lipofectamine RNAiMAX (Invitrogen, Grand Island, NY, USA). The cells were then harvested after 3 days, and the mRNA expression levels were analyzed.

### 4.3. Mice

The mice were maintained and anesthetized according to a protocol approved by the United States Pharmacopeia and the Institutional Animal Care and Use Committee of Yonsei University (IACUC-201902-866-01; 11 March 2019).

### 4.4. RNA Extraction and qRT-PCR

Total RNA was extracted from dermal papilla cells, outer root sheath cells, and mouse dorsal skin tissues using Invitrogen TRIzol Reagent (Thermo Fisher Scientific). cDNA synthesis was performed using oligo dT and the HelixCript Thermo Reverse Transcription System (NanoHelix Co., Ltd., Daejeon, South Korea) according to the manufacturer’s instructions. For the qRT-PCR reaction, BrightGreen qPCR master mix-ROX (abm, New York, NY, USA) was used. The *gapdh* mRNA expression level was used for sample standardization. 

### 4.5. Growth Factor Polymerase Chain Reaction Array

Growth factor expression profiles of hDPCs or hORS cells were analyzed using RT² Profiler PCR Array (PAHS-041Z; Qiagen, Hilden, Germany).

### 4.6. Western Blot Analysis

For tissue sampling, the dorsal skin tissues of anagen mice and telogen mice were rapidly frozen using liquid nitrogen and ground and were then lysed with protein extraction solution (PRO-PREP^™^; iNtRON, Seoul, South Korea). Protein quantification was performed using the BCA analysis kit (Pierce BCA Protein Assay Kit; Thermo Fischer, Hanover, IL, USA), and then 50 µg of protein was run on a 12% acrylamide gel for 1.5 h. All proteins were transferred to the polyvinylidene fluoride membrane (Immobilon-P; Merck Millipore, Darmstadt, Germany) and blocked with 5% bovine serum albumin blocking buffer for 1 h at room temperature. Subsequently, the membrane was incubated with Rab27a primary antibody (Cell Signaling, Danvers, MA, USA; 1:1000; #Cat. 69295S), Rab27b primary antibody (Proteintech Group, Rosemont, IL, USA; 1:1000; #Cat. 13412-1-AP), or mouse α-tubulin (Santa Cruz Biotechnology, Dallas, TX, USA; #Cat. sc-32293) overnight at 4 °C. The membrane was then washed three times with tris-buffered saline with Tween 20 and incubated with secondary antibodies (1:2000) for 1 h at room temperature. The secondary antibodies used were peroxidase-labeled anti-mouse IgG (PI-2000; Vector Laboratories, Burlingame, CA, USA) and peroxidase-labeled anti-rabbit IgG (PI-1000, Vector Laboratories). After adding enhanced chemiluminescence solution (Immobilon Western, Millipore, Burlington, MA, USA), western blot images were obtained using ImageQuant LAS 4000 (GE Healthcare Life Science, Bensalem, PA, USA).

### 4.7. Immunofluorescence Staining

For immunofluorescence staining, paraffin sections were melted in an incubator at 65 °C for 18 min and were then dewaxed three times for 10 min in xylene. After this, the sections were dehydrated twice in 100% ethyl alcohol and once in 90%, 80%, and 70% ethyl alcohol. Sections were boiled in boiling antigen retrieval solution (pH 6.0; Dako, Carpinteria, CA, USA) for 2 min and 20 s and then cooled. These sections were stained overnight at 4 °C with Rab27a primary antibody (Cell Signaling; 1:200), Rab27b primary antibody (Proteintech Group, Rosemont, IL, USA; 1:200), and giantin for Golgi marker [31] (Abcam; 1:200). The next day, these sections were stained with Alexa Fluor 488 goat anti-rabbit IgG (Invitrogen; 1:1000) or Alexa Fluor 594 goat anti-mouse IgG (Invitrogen, Grand Island, NY, USA; 1:1000) for 1 h at room temperature with 4,6-diamidino-2-phenylindole (Sigma-Aldrich, St. Louis, MO, USA). Immunofluorescence staining was imaged using a Zeiss LSM700 confocal microscope, with 30× magnification.

### 4.8. Treatment of Growth Factors Cocktail

In experiments using hDPCs or hORS cells, the growth factors cocktail was treated at a concentration of 0.01 ng/mL or 0.1 ng/mL. In the organ culture, the growth factors cocktail was treated at a concentration of 10 ng/mL. The composition of the growth factors cocktail was as follows: recombinant human PIGF-2 (PGF; PeproTech, Rocky Hill, NJ, USA), recombinant human pleiotrophin (PTN; PeproTech), recombinant human GRO-α/MGSA (CXCL1; PeproTech), recombinant human midkine (MDK; PeproTech), recombinant human FGF-23 (PeproTech), recombinant human IL-4 (PeproTech), recombinant human bone morphogenetic protein BMP-10 (PeproTech), and recombinant human G-CSF (CSF3; PeproTech). 

### 4.9. Cell Proliferation Assay

hDPCs or hORS cells were seeded in six-well plates at a density of 1.5 × 10^4^ per well and were treated with Rab27a siRNA (1, 10 or 100 nM), Rab27b siRNA (1, 10 or 100 nM), growth factors cocktail (0.01 or 0.1 ng/mL), or Nexinhib20 (2 or 20 µM) for 3 days. The proliferation of cells was then measured using a Cell Counting Kit-8 (Dojindo Molecular Technologies, Inc., Rockville, MD, USA) according to the manufacturer’s instructions.

### 4.10. Scratch Migration Assay

To measure the migration ability of the cells, a six-well plate filled with hDPCs or hORS cells was scratched with a constant width and then washed with phosphate-buffered saline (PBS). Thereafter, scramble siRNA, Rab27a siRNA, or Rab27b siRNA was treated for 24 h in the normal medium at a concentration of 1, 10, or 100 nM, respectively. The migration ability of the cells was analyzed by how many cells were filled into the scratched area, and the image was visualized using a ZEISS Observer D1 microscope.

### 4.11. Organ Culture

The vibrissae follicles of 4-week-old female C57BL/6J mice were isolated from the specimen using scalpel and forceps. The experiment was conducted according to the method described by Jindo et al. [32]. The scramble siRNA, Rab27a siRNA, or Rab27b siRNA was treated with 6 µg of siRNA per 300 µl of the medium. At the same time, it was treated with delivery reagent (in vivo-jetPEI; Polyplus-Transfection, New York, NY, USA) to ensure the siRNA was well delivered. The length of the grown hair shaft of mice was then calculated using ImageJ software (version 1.45; National Institutes of Health, Bethesda, MD, USA) after 2 or 3 days. The HFs of pig skin were trimmed and cultured in the same way as in mice, and the length of the grown hair was calculated after 5 days.

### 4.12. Anagen Induction

The mice used in the experiment were 6-week-old male C3H mice entering the telogen phase, and these were mice between 36 and 42 days old. In order to reduce the difference between mice, when performing a set of experiments, the difference in the birth date of each mouse is usually limited to within 2–3 days, and the weight difference is limited to within 2 g. Therefore, it is assumed that mice in all groups have the same initial conditions in one experimental set. However, because each different set of experiments was performed independently, the initial conditions of the mice between each set of experiments may not be completely consistent. The dorsal hair of mice in the telogen phase was shaved with an electric shaver, and scrambled siRNA, Rab27a siRNA, or Rab27b siRNA was injected into the dorsal skin of mice shaved three times, once every 3 days by subcutaneous injection. At this time, each siRNA was injected (12 μg) with the delivery reagent (in vivo-jetPEI; Polyplus-Transfection). The detailed method is as follows. The siRNA was diluted into 1/2 the injection volume in 5% glucose (final concentration) using the 10% glucose stock solution and sterile water. In vivo-jet PEI reagent was diluted into 1/2 the injection volume in 5% glucose (final concentration) using the 10% glucose stock solution and sterile water. Thereafter, the diluted in vivo-jet PEI reagent was added to the diluted siRNA and incubated for 15 min at room temperature. And it was injected into mice. After 16 days, the grown hair and dorsal tissues in the shaved area of the Rab27a siRNA-treated mice were harvested. The grown hair and dorsal tissues in the shaved area of the Rab27b siRNA-treated mice were harvested 14 days later. The skin darkening of the mice and the weight of the harvested hair were used to evaluate the hair growth rate.

### 4.13. Hematoxylin and Eosin Staining

The paraffin sections of the harvested mouse tissues were dewaxed in xylene for 30 min and then dehydrated sequentially in 100%, 90%, 80%, and 70% ethyl alcohol. The tissue slides were rinsed for 1 min with water, soaked in Mayer’s hematoxylin (Sigma-Aldrich) for 10 min, and washed three times with water. The slides were then dipped in eosin Y (Sigma-Aldrich) for 90 s and washed three times with water. They were then dehydrated with 70%, 80%, 90%, and 100% EtOH, washed again with xylene for 20 min, dried, and mounted with mounting medium (Sigma-Aldrich). Hematoxylin and eosin staining was used to assess the hair cycle phase of the hair and to quantitatively evaluate the anagen phase of the HFs.

### 4.14. Statistical Analysis

Data were presented as the means from three independent experiments. Student’s *t* test was used between two groups. Analysis of variance, followed with Tukey’s post hoc test, was used for comparing multiple groups. The results were presented as the means ± standard error of the mean. A *p* value of less than 0.05 was considered to indicate a statistically significant difference. All statistical analyses used GraphPad Prism 5.01 (GraphPad Software Inc., San Diego, CA, USA).

## Supplementary Materials

Supplementary materials can be found at https://www.mdpi.com/1422-0067/21/16/5672/s1.

## Abbreviations

hDPCs	Human dermal papilla cells
hORS cells	Human outer root sheath cells
Mx	Matrix cells
SG	Sebaceous gland
GF	Growth factor
*MDK*	Midkine
*CXCL1*	chemokine (C-X-C motif) ligand 1
*PTN*	Pleiotrophin
*IL-4*	Interleukin-4
*CSF3*	Colony Stimulating Factor 3
*PGF*	Placental growth factor
*FGF-23*	Fibroblast growth factor 23
*BMP-10*	Bone morphogenetic protein 10
*CLC*	Charcot-Leyden crystal protein
*NTF3*	Neurotrophin 3
*GDF10*	Growth differentiation factor 10
*NRG2*	Neuregulin 2
*BMP4*	Bone morphogenetic protein 4
*B2M*	Beta-2-microglobulin
*IGF1*	Insulin-like growth factor 1 (somatomedin C)
