# Supplementary material for: Inhibition of Rab27a and Rab27b Has Opposite Effects on the Regulation of Hair Cycle and Hair Growth
Source: Int J Mol Sci. 2020 Aug 7;21(16):5672. doi: 10.3390/ijms21165672 (PMC7460818; doi:10.3390/ijms21165672)
Supplement: Supplementary file 1 [file ijms-21-05672-s001.pdf]

Supplemental Figure 1.

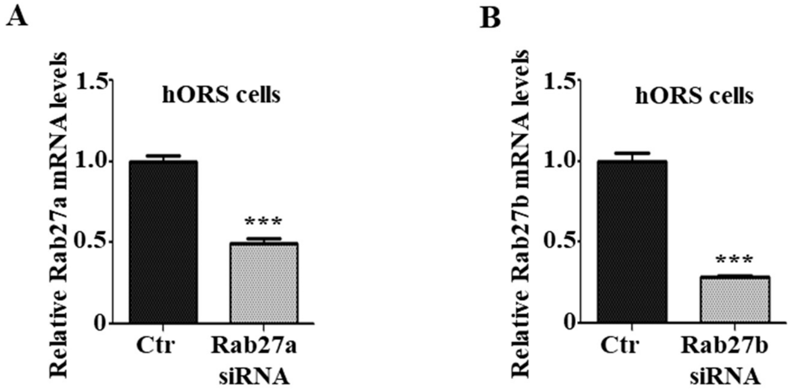

Supplemental Figure 1. Inhibition effects of Rab27a/b siRNA in hORS cells. (A) In hORS cells, Rab27a siRNA inhibited Rab27a expression by approximately 50%. (B) Rab27b siRNA inhibited the expression of Rab27b by approximately 70%. *** $p < 0.001$ using Student's *t*-test. Three independent experiments were conducted. All error bars indicate S.E.M.

Supplemental Figure 2.

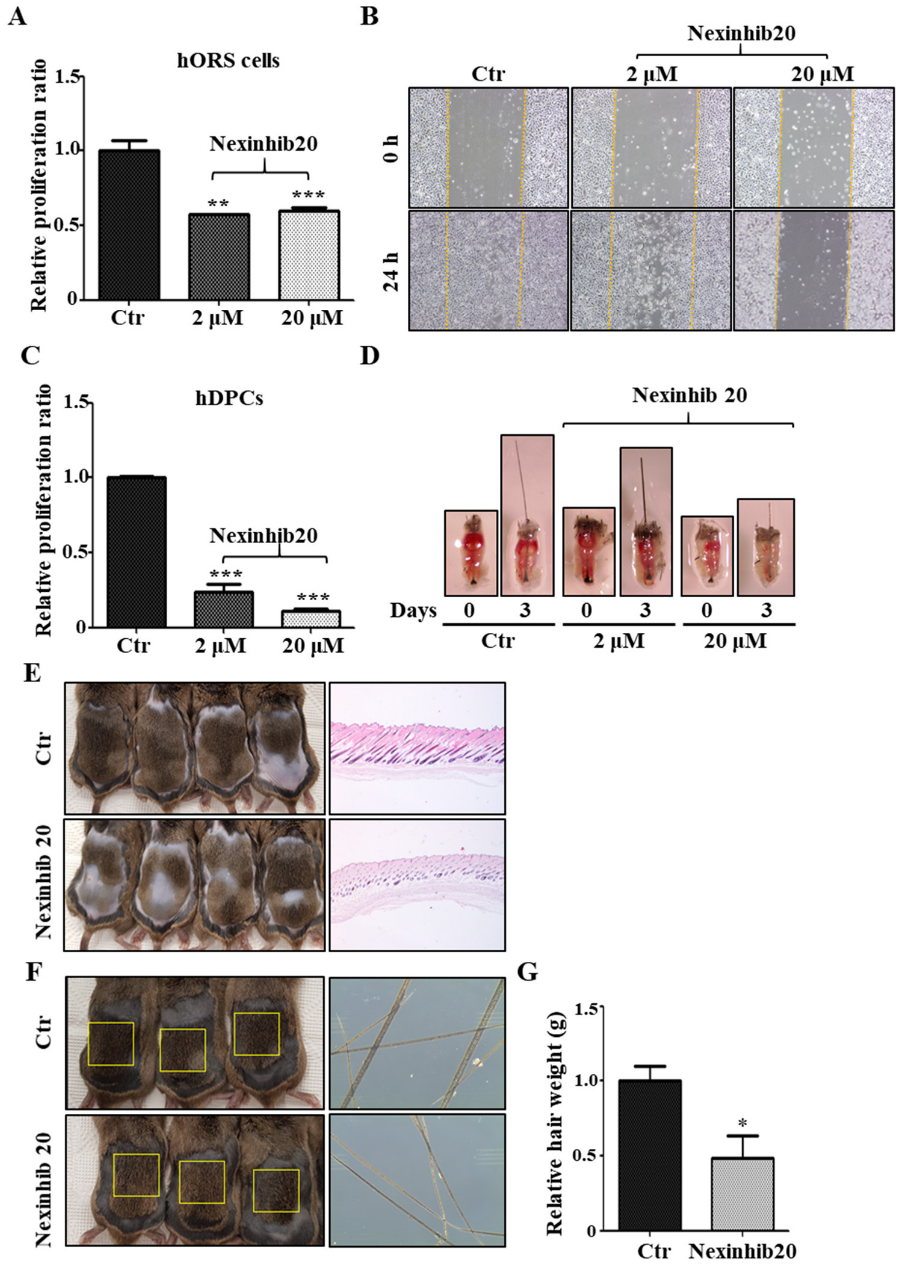

Supplemental Figure 2. Nexinb20, a Rab27a inhibitor, inhibited the mitogenic effects of hORS cells and hDPCs, as well as hair growth. (A) Nexinb20 inhibited the proliferation of hORS cells and (B) migration. (C) The proliferation of hDPCs was also inhibited. (D) Nexinb20 suppressed mouse vibrissae hair growth in a dose-dependent manner. (E) The surrounding skin injected with Nexinb20 interfered with the telogen-to-anagen transition. (F) Nexinb20 induced thinner, brighter hair than controls. (G) Accordingly, the weight of the hair was also light. * $p < 0.05$, ** $p < 0.01$, *** $p < 0.001$ using Student's *t* test. Three independent experiments were conducted. All error bars indicate standard error of the mean.

Supplemental Figure 3.

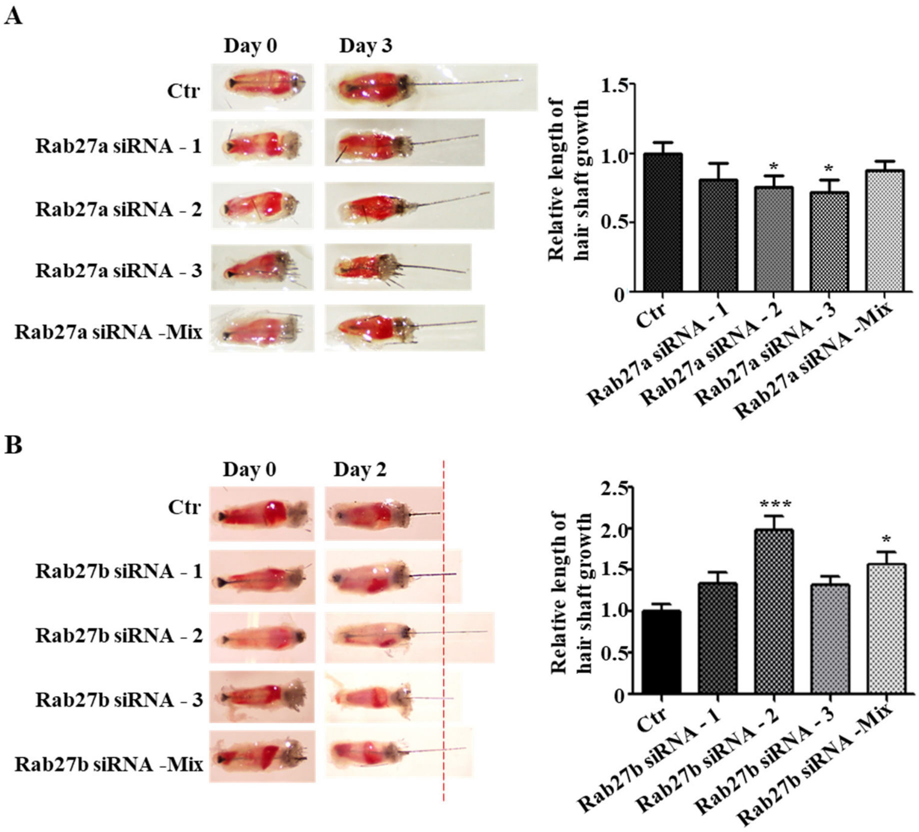

Supplemental Figure 3. Inhibition of Rab27a or Rab27b using several siRNAs had an opposite effect on vibrissae hair growth in mice. (A) Rab27a inhibition using three sequences of Rab27a siRNA inhibited the growth of vibrissae hair in mice. (B) Three sequences of Rab27b siRNA promoted the growth of vibrissae hair in mice. The Rab27b siRNA-2 sequence showed the greatest effect on hair growth. * $p < 0.05$, *** $p < 0.001$ using ANOVA followed with Tukey's post hoc test or Student's *t*-test. Three independent experiments were conducted. All error bars indicate standard error of the mean.

Supplemental Figure 4.

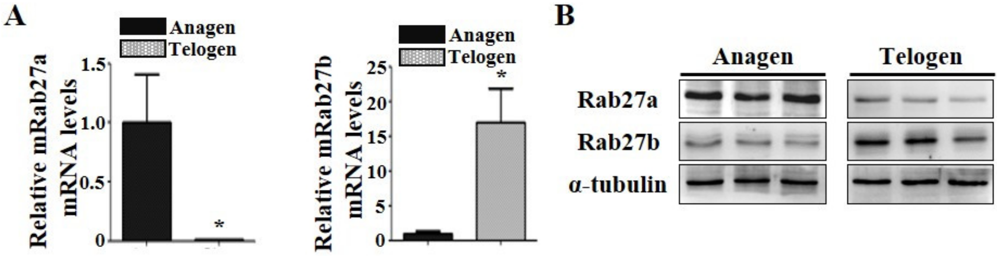

Supplemental Figure 4. The expression of Rab27a and Rab27b showed opposite patterns in the hair cycle of normal C3H mice. (A) In mRNA obtained from the dorsal skin of normal C3H mice, Rab27a mRNA had a high expression in the anagen phase and low expression in the telogen phase. On the other hand, the expression of Rab27b mRNA was very high in the telogen phase compared with the anagen phase. (B) Similarly, Rab27a protein was highly expressed in the anagen phase, and Rab27b protein was highly expressed in the telogen phase. * $p < 0.05$ using Student's *t*-test. Three independent experiments were conducted. All error bars indicate S.E.M.
